# Healthy Lifestyle and Cancer Risk: Modifiable Risk Factors to Prevent Cancer

**DOI:** 10.3390/nu16060800

**Published:** 2024-03-11

**Authors:** Pasquale Marino, Mariangela Mininni, Giovanni Deiana, Graziella Marino, Rosa Divella, Ilaria Bochicchio, Alda Giuliano, Stefania Lapadula, Alessandro Rocco Lettini, Francesca Sanseverino

**Affiliations:** 1 Unit of Oncological Gynecology, Centro di Riferimento Oncologico della Basilicata (IRCCS-CROB), Via Padre Pio, 1, 85028 Potenza, Italy; giovanni.deiana@crob.it (G.D.); francesca.sanseverino@crob.it (F.S.); 2 Department Direzione Generale per la Salute e le Politiche della Persona, Regione Basilicata, Via Vincenzo Verrastro, 4, 85100 Potenza, Italy; mariangela.mininni@regione.basilicata.it; 3 Unit of Breast Surgery, Centro di Riferimento Oncologico della Basilicata (IRCCS-CROB), 85028 Potenza, Italy; graziella.marino@crob.it; 4 Nutritionist’s Studio at the Gravina in Puglia, C.so Giuseppe Di Vittorio, 14, 70024 Bari, Italy; rosadive@inwind.it; 5 Unit of Clinical Psychology, Centro di Riferimento Oncologico della Basilicata (IRCCS-CROB), Via Padre Pio, 1, 85028 Potenza, Italy; ilaria.bochicchio@crob.it (I.B.); aldagiuliano@gmail.com (A.G.); stefania.lapadula@crob.it (S.L.); alessandro.lettini@crob.it (A.R.L.)

**Keywords:** healthy lifestyle, cancer risk, cancer prevention recommendations, breast cancer, colorectal cancer, prostate cancer, modifiable risk factors

## Abstract

Cancer has become a serious problem worldwide, as it represents the main cause of death, and its incidence has increased over the years. A potential strategy to counter the growing spread of various forms of cancer is the adoption of prevention strategies, in particular, the use of healthy lifestyles, such as maintaining a healthy weight, following a healthy diet; being physically active; avoiding smoking, alcohol consumption, and sun exposure; and vitamin D supplementation. These modifiable risk factors are associated with this disease, contributing to its development, progression, and severity. This review evaluates the relationship between potentially modifiable risk factors and overall cancer development, specifically breast, colorectal, and prostate cancer, and highlights updated recommendations on cancer prevention. The results of numerous clinical and epidemiological studies clearly show the influence of lifestyles on the development and prevention of cancer. An incorrect diet, composed mainly of saturated fats and processed products, resulting in increased body weight, combined with physical inactivity, alcohol consumption, and smoking, has induced an increase in the incidence of all three types of cancer under study. Given the importance of adopting correct and healthy lifestyles to prevent cancer, global institutions should develop strategies and environments that encourage individuals to adopt healthy and regular behaviors.

## 1. Introduction

Cancer is a leading cause of death worldwide and represents a growing major public health problem. The data collected reported that, in 2020, there were 19.3 million new cases and 10 million deaths from cancer worldwide. Breast cancer is the most commonly diagnosed cancer in the world, followed by lung cancer. Prostate cancer was the most frequently diagnosed cancer in males, followed by lung cancer, non-melanoma skin cancer (NMSC), lip and oral cavity cancer, and liver cancer [1]. Moreover, breast cancer is the most commonly diagnosed cancer in women worldwide, accounting for 11.7% of all total cases and with 2.3 million new cases. Breast cancer is the fifth leading cause of cancer-related death in the world, with a mortality rate of 685,000 deaths and 6.9% of total cancer deaths [2].

In recent years, several strategies have been explored to fight cancer in addition to surgery, radiotherapy, and chemotherapy. It is well known that the onset of most types of cancer is preventable through the adoption of a healthy lifestyle, including avoiding smoking, maintaining a healthy weight, exercising, eliminating harmful alcohol use, and eating a healthy diet. This type of strategy is defined as “best buy” and prevents and manages cancer and other non-communicable diseases [3]. Conversely, unhealthy lifestyles, including exposure to tobacco smoke, alcohol consumption, high body mass index (BMI), physical inactivity, and poor dietary customs, are linked to an increased risk of cancer [4]. Current evidence shows that, by changing behavior in relation to modifiable risk factors, such as diet, large numbers of cancer cases can be prevented [5]. Numerous studies show a potential connection between eating styles with certain types of cancer, such as breast [6], gastric [7], and colorectal cancer [8].

In this review article, we examine the current evidence on how lifestyle importantly influences various types of cancer, particularly breast, colorectal, and prostate cancer.

### 1.1. Healthy Lifestyle Factors

**Figure 1:**
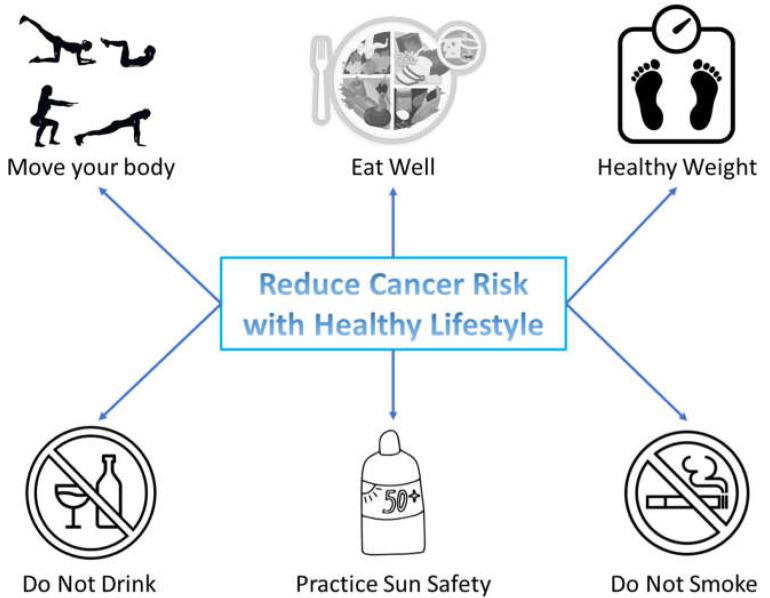
Healthy lifestyle for the prevention of cancer.

The World Health Organization (WHO) defines health as physical, mental, and social well-being, not just the absence of disease. To achieve this state, it is necessary to control, organize, and choose all the behaviors that can have repercussions on an individual’s health. This type of health development and management is called a healthy lifestyle (Figure 1), which includes not only protection from disease but also the use of behaviors that increase well-being throughout life [9]. Behaviors such as proportional and acceptable food, stress control, reasonable and constant physical activities, and elimination of tobacco are among the essential elements of a healthy lifestyle [10].

In 2018, the World Cancer Research Fund (WCRF) and the American Institute for Cancer Research (AICR) released a report supporting updated cancer prevention recommendations (Table 1). The report, “Diet, Nutrition, Physical Activity and Cancer: A Global Perspective”, sets out a series of lifestyle recommendations, including following a healthy diet, preserving a normal BMI, and committing to staying physically active. The report highlights that the recommendations are a series of adaptable behaviors that facilitate the prevention of cancer, other non-communicable diseases, and obesity through consistent exercise and a healthy, balanced diet [11].

In addition to these recommendations, not participating in active or passive smoking and not exposing yourself excessively to the sun is also essential to decreasing cancer risk [12]. It is known that smoking significantly enhances the risk of various tumors, in particular, lung cancer, but also oral cavity, pharynx, larynx, and esophagus cancer. The most important strategy for preventing cancer is smoking prevention, according to which, people who never smoke or who quit smoking have a much-reduced chance of death in the next 15 years compared with those who continue to smoke [13,14].

A much-debated topic among researchers is the relationship between exposure to the sun and the risk of developing diseases, in particular, skin diseases such as rashes and especially cancer. Exposure to solar radiation has been shown to have many adverse health effects, as it generates changes at the molecular and cellular level, such as DNA damage. There are many variables regarding the action of UV rays. Among the most important variables, behavioral habits, such as exposure times and use of protective creams, and environmental factors, such as latitude and the angle of the rays on the affected body region, produce more significant effects [15]. Studies have shown that solar radiation induces melanoma, squamous cell carcinoma of the skin, and basal cell carcinoma of the skin. Conversely, numerous other studies highlight that exposure to sunlight is of fundamental importance and produces numerous beneficial effects for human health [16]. In fact, the data have revealed that a lack of exposure to sunlight leads to the insufficient production of vitamin D, with a consequent increase in the risk of developing diseases, including cancer. It has been shown that high serum 25-hydroxyvitamin D (25(OH)D) concentrations in circulation reduce the risk of developing erythema, skin cancer, and melanoma, while low serum concentrations, below optimal levels, come with a greater risk of developing the disease [17,18]. The consequent low sun exposure following skin cancer prevention campaigns leads to vitamin D deficiency with a subsequent loss of the benefits associated with it. It is clear that a greater balance in sun exposure is necessary in order to reap the benefits and limit damage [19]. A possible strategy to increase serum levels of 25(OH)D comes from dietary intake, particularly in red meat, fatty fish, salmon, tuna, mackerel, milk, and dairy products, which have high amounts of vitamin D [20,21].

These guidelines aim to reduce the incidence of cancer by allowing people to preserve a healthy weight and eat a correct and balanced diet; induce greater physical exercise; and reduce or eliminate alcohol consumption for life. These recommendations are designed to be used by diverse groups including individuals, families, healthcare workers, and communities.

Numerous studies have highlighted the difference between adhering to a single healthy behavior and the combination of multiple behaviors on the quality and length of life of individuals [22,23,24]. A recent study on the effectiveness of physical exercise alone on the well-being of breast cancer survivors has highlighted that adhering to just one healthy behavior is not adequate, but that to have a greater state of well-being, patients should be induced to follow more healthy lifestyle behaviors [25]. This evidence was even demonstrated by further analysis, which highlighted a linearity between adherence to multiple recommended healthy lifestyle factors and health benefits. For this reason, compliance with single behaviors is unlikely to have a positive effect on the quality and longevity of life [26]. Further cohort studies have shown that men and women who have an adequate body weight and observe three or more regular lifestyle habits have a mortality rate decreased by approximately 60% [27,28].

### 1.2. Life’s Essential 8 and Cancer

An additional parameter used to evaluate the factors within a healthy or unhealthy lifestyle is based on the use of Life’s Simple 7, subsequently updated in 2022 with Life’s Essential 8, as proposed by the American Heart Association. This is a method based on the measurement of cardiovascular health (CVH) that evaluates four health behaviors, diet, physical activity, nicotine exposure, and sleep health, and four health factors, body mass index, blood lipids, blood sugar, and blood pressure. These scores allow us to evaluate how lifestyle significantly influences cardiovascular health and the risks of death from all causes, including cancer, for individuals who adhere to Life’s Essential 8. For each parameter of Life’s Essential 8, a scale of 0–100 is assigned, and the average of all parameters represents the final cardiovascular health score. CHV is divided into three levels: a high or optimal CHV level from 80 to 100; a moderate CHV level from 50 to 79; and finally, a low CHV level from 0 to 49 [29,30,31,32].

Numerous studies have highlighted how healthy lifestyle behaviors with high CVH scores are associated with a lower risk of mortality, while low CVH scores are linked to higher risks of all-cause, cancer-specific, and non-cancer mortality. A study carried out by Fun et al. in 2024 highlighted that, in a group of patients with high CVH, in the 15-year follow-up, the mortality rate was lower than the mortality rate that was found in the group with low CVH. Furthermore, in a subgroup study, the association between Life’s Essential 8 and the socioeconomic conditions of the patients in the risk of mortality from all causes including cancer was evident [30]. Another study showed that patients with high CVH had a 40% reduction in the risk of death from all causes, including cancer, and a 54% reduction in the risk of death from cardiovascular problems, compared with patients with low CVH [33]. Furthermore, it was seen that patients with high CVH had a higher disease-free life expectancy by an average of 5.2 years among men and 6.3 years among women compared with patients with low CVH [34].

These data highlight how the behaviors and lifestyles that individuals adopt are of fundamental importance for a person’s health and life expectancy.

## 2. Healthy Lifestyle and Cancer

Cancer is recognized worldwide as the leading cause of death for individuals under the age of 85 or, overall, as the second leading cause of death, preceded only by cardiovascular disease [35]. It is a complex disease and is the result of genetic and epigenetic changes that accumulate and lead healthy cells to transform. There are three main stages that induce cancer formation, namely, initiation, promotion, and progression. The initiation phase is an irreversible and very rapid event in which an alteration of the cell’s DNA occurs that causes the cell to become cancerous. This genotoxic damage is due to various causes: it can be due to a spontaneous mutation or be the result of the action of endogenous or exogenous carcinogens. These etiological agents induce the activation of so-called oncogenes or the inactivation of tumor suppressor genes. The second phase of the transformation process is called promotion. In this phase, the preneoplastic cell undergoes the action of agents, such as growth factors, hormones, and UV radiation, which promote its proliferation and enable transformation. This means that daughter cells present a higher number of mutations within their already-transformed genetic material. Finally, the progression phase occurs, in which uncontrolled cellular proliferation, greater aggressiveness and invasiveness, and greater metastatic power occur, determining the definitive neoplastic transformation [36].

The severity of this disease goes beyond its mortality rate; in fact, patients throughout the entire phase of the disease experience physical and psychological pain and a general reduction in the quality of life. Life is turned upside down by anti-tumor therapies, by the short and long-term adverse effects of treatment, and by the countless diagnostic procedures to which they are subjected. Furthermore, quality of life can also be substantially compromised for those close to patients [37].

### 2.1. Overweight and Obesity

Obesity and high BMI represent a fundamental factor, second only to smoking, as the most common cause of cancer [38] and increase the risk in both men and women [39]. A study of adults in the United States highlighted that obesity is responsible for 5% of cancer cases in men and 10% of cases in women [40,41]. Numerous studies have explained the mechanisms according to which body weight and, in particular, obesity induce an increased risk of developing various types of cancer. Excessive body weight has been shown to induce imbalances within adipose tissue, promoting the release of cytokines such as IL-6 and TNF. Cytokines cause DNA damage, induce point mutations, promote cell proliferation, and induce angiogenesis and invasiveness of tumor cells, leading to the development and progression of cancer [42].

Furthermore, adipose tissue being in a state of imbalance promotes a state of chronic inflammation, which leads to an increased risk of cancer [43]. Another important mechanism linked to obesity is a cellular environment with high oxidative stress and high reactive oxygen species (ROS) production that damages DNA. The excess of ROS also contributes to carcinogenesis because of the low consumption of a healthy diet rich in antioxidants [44,45,46]. An additional mechanism underlying the development of various forms of cancer is also the unregulated production of hormones such as estrogens, androgens in women, leptin, and growth hormones [47,48]. Studies have also shown that reducing body weight can help individuals prevent cancer and improve treatments [49]. Other work has shown that obesity is not only linked to the risk of developing cancer but also increases the risk of recurrence and mortality among patients who have defeated cancer [50,51]. This is why it is essential to intervene in nutritional education to prevent the development of various types of cancer.

Numerous studies have highlighted that weight gain in adulthood is related to various types of cancer such as breast, colorectal, prostate, kidney, ovarian, stomach, endometrial, esophageal, pancreatic, and other cancers [52,53].

### 2.2. Diet

Diet and nutrition represent one of the most important factors in the development of cancer; in particular, a correct diet, such as the Mediterranean diet, rich in fruit, vegetables, and fiber, is often associated with a reduction in the risk of the onset of cancer. Conversely, a diet rich in saturated fats, red meat, and derivative products can increase the risk of developing cancer, consequently increasing its incidence [37,54]. It has been shown that eating a healthy diet can prevent the development of cancer by 30–50% [55]. Numerous studies have highlighted that the regular consumption of fruits and vegetables rich in bioactive components such as polyphenols and carotenoids but also rich in vitamin B12, folic acid, and selenium, as well as the intake of foods rich in fiber, milk, and dairy products, has a preventive role with respect to various types of cancer, such as colorectal, breast, stomach, prostate, and esophageal cancer [56,57,58]. In addition to these, omega-3s, contained in large quantities in oily fish and nuts, are also of fundamental importance in reducing the risk of the onset of this disease [59].

Putting all this information together, it is clear that the Mediterranean diet is the best dietary model among the various types of diet since it is the one that comes closest to the ideal healthy diet [60,61]. Numerous studies have shown that one of the mechanisms underlying the protective effect of the Mediterranean diet is represented by the antioxidant and anti-inflammatory action of the numerous bioactive components contained in foods capable of inducing apoptosis, reducing inflammation, inhibiting cell proliferation, and reducing angiogenesis and invasiveness of tumor cells [62,63,64]. Furthermore, it has been demonstrated by numerous studies that there is an inverse relationship between the regular and constant use of the Mediterranean diet and the risk of mortality and the incidence of various types of cancer such as colorectal, breast, prostate, liver, stomach, and head and neck cancer [65,66,67].

### 2.3. Vitamin D

Vitamin D belongs to a class of fat-soluble steroid hormones involved in numerous physiological processes, including the regulation of calcium and phosphorus homeostasis and bone mineralization [68]. Furthermore, numerous discoveries have highlighted numerous biological properties, outside of bone metabolism, including inhibiting tumor cell growth, angiogenesis, and cell metastasis and inducing apoptosis in various tumor types [69]. Numerous studies have shown that lower serum 25(OH)D levels are linked to a higher risk of developing various types of tumors, while high 25(OH)D levels have been found to have a protective effect against the development of cancer [70,71]. Furthermore, taking a vitamin D supplement in patients diagnosed with cancer has been linked to a favorable prognosis [72,73,74]. Observational studies have demonstrated a strong relationship between low levels of circulating 25(OH)D and a higher risk of developing colorectal, breast, and prostate cancer [75,76,77,78,79,80]. For prostate cancer, there are still conflicting studies in which low or no association has been highlighted between vitamin D and the risk of prostate cancer [81].

A factor that could suggest a decisive function of vitamin D in the development and advancement of cancer is the presence of the specific receptor for vitamin D on malignant cells and not only on bone cells or cells responsible for calcium regulation [81,82]. There are numerous mechanisms through which vitamin D carries out its anti-tumor actions. Vitamin D has been shown to be important in tumor suppression by blocking cell proliferation; inducing apoptosis, autophagic death, and anti-inflammatory action; and inhibiting angiogenesis, cell invasiveness, and metastasis [83]. These actions are probably due to the ability of vitamin D to regulate intracellular calcium, influencing the pathways involved in cell growth and apoptosis [84].

### 2.4. Physical Activity

The preventive role of exercise on cancer is now known. In particular, numerous data demonstrate that physical activity reduces the risk of various types of cancer, such as bladder, breast, colorectal, endometrial, esophageal, and stomach cancer, but also a reduction in the incidence of lung, kidney, pancreatic, and ovarian cancer [85]. One study found that increased physical exercise is linked to a 10–25% reduction in the risk of developing cancer [86]. A further study highlighted that intense physical exercise for at least 15-20 min per week was linked to a reduction in mortality risk of 16–40%, with a more marked reduction for physical activity of 50–57 min per week [87]. Other epidemiological studies have shown that intense and vigorous physical exercise compared with a sedentary lifestyle lowers the risk of death by 64%. At the same time, reduced mortality risks of 44% and 42% have been found for moderate and mild exercise, respectively. Even in cancer survivors, a regular lifestyle with good physical activity represents increased survival and quality of life for patients [88,89].

Among the mechanisms underlying the positive effect of physical exercise, the most important appears to be the ability of exercise to influence the immune system and inflammation. Moderate, regular physical activity has been shown to activate the immune system and reduce inflammation, while a sedentary lifestyle suppresses the immune system, induces inflammation, and increases the risk of developing cancer. Lack of physical exercise leads to increasing fat in adipose tissue and the activation of inflammatory factors that cause chronic inflammation, which is also responsible for tumor growth [85,90].

### 2.5. Alcohol Consumption

Alcohol is becoming a primary public health problem worldwide. Alcohol is considered to be a very toxic substance and, as far as cancer is concerned, a Group 1 carcinogen responsible for various types of cancer, including esophageal, liver, colorectal, and breast cancer [91]. It has been estimated that alcohol is responsible for approximately 4% of cancers worldwide, equating to over 740,000 cases/year [92]. The main types of cancer resulting from alcohol consumption are esophageal, liver, and breast cancer. For these reasons, one of the global objectives is to improve the prevention of alcohol consumption. The results of studies on alcohol consumption present conflicting results. Some studies highlight the ability of alcohol, for even moderate consumption (one or two glasses a day), to increase the risks of developing breast, colorectal, liver, and esophagus cancer and oral cavities [93,94,95], while other studies have shown how moderate consumption, between 6 and 25 g/day, reduces the risks of developing cancer [96]. A study in the United States showed that cancer risks were lower in patients who consumed low amounts of alcohol compared with non-drinkers [97]. In support of these data, studies carried out in countries that use the Mediterranean diet as an eating style have revealed that the moderate consumption of red wine during meals is an important factor in reducing the risk of developing cancer and protecting against cardiovascular diseases [98,99]. The responsibility for these phenomena could be attributed to the large quantity of polyphenols present in red wine, which exert their antioxidant and anti-inflammatory properties [100,101].

Numerous studies have attempted to determine the mechanisms and molecular pathways of cancer development linked to alcohol consumption. Alcohol has been shown to induce increased oxidative stress and inflammation in cancer cells leading to further damage to genetic material. Furthermore, it has been shown that, particularly in breast cancer, the harmful properties of alcohol promote cell proliferation and angiogenesis and increase the abilities of tumor cells to invade and metastasize to other tissues [102].

### 2.6. Smoking

Cigarette smoking is still considered one of the most significant, if not the most significant, risk factors in the development of cancer. It is considered one of the most significant causes of mortality [103]. The relationship between cigarette smoking and lung cancer has been widely demonstrated [104,105]. There is also significant evidence that has highlighted the strong relationship between smoking and cancer of the bladder, head and neck, stomach, colorectal, esophagus, pancreas, kidney, liver, and cervix [106,107,108]. Furthermore, other studies have shown that individuals who smoke continuously have a greater risk of developing diseases such as cancer and chronic cardiovascular and inflammatory disorders [109,110,111]. It has also been shown that patients who smoke even after a cancer diagnosis have a higher risk of mortality due to smoking-related respiratory and cardiovascular problems but also a higher possibility of tumor recurrence, complications during treatment, and a risk of developing new primary tumors [112,113].

The mechanism underlying the action of smoking on the development of cancer is the presence of a complex mixture of carcinogenic compounds, including polycyclic aromatic hydrocarbons (PAHs), nitrosamines, and acrylamides capable of inducing DNA damage with consequent bricking and cancer development. Furthermore, smoke also contains numerous reactive oxygen and nitrogen species, which create an environment with high oxidative stress capable of damaging molecular targets such as DNA, lipids, and proteins and inducing a state of chronic inflammation, which are proven causes of cancer development [106,114].

## 3. Healthy Lifestyle and Breast Cancer

Breast cancer is the cancer with the highest rate of diagnosis in women and involves approximately 13% of women in highly developed countries. Furthermore, it is still the leading reason for cancer-related death for women [35,115]. It has been shown that, in different regions of the world, the incidence, mortality, and survival rates of breast cancer vary greatly. In more developed countries, the incidence of breast cancer is increasing not only because of an increase in mammographic screening, which results in a greater number of diagnoses but also incorrect adherence to prevention behaviors [116,117]. At the same time, breast cancer mortality is decreasing thanks to early diagnosis and improved treatments [116]. Breast cancer is known to be a multifactorial disease and is caused by various factors such as population structure, lifestyle, genetic factors, and environment [118,119]. Prevention presents itself as the best strategy to reduce the risk of disease development, conducted, in particular, by adhering to healthy lifestyle behaviors.

### 3.1. Overweight and Obesity

Numerous analyses have highlighted the association between overweight, obesity, and breast cancer [120,121]. Obesity is believed to be connected to breast cancer because adipose tissue, particularly in postmenopausal women, is the main font from which estrogens are released, which increases the risk of developing a tumor [122,123]. In a meta-analysis of numerous studies, there was a clear difference between postmenopausal women who had a high body weight and those who had a lower body weight; women with higher weight had an 82% increased relative risk (RR) of developing ER-positive breast cancer [124]. Moreover, an additional study of 74,177 women found that weight gain in young women was responsible for 17% of ER+/PR+ postmenopausal breast cancer cases and, overall, 14% of total postmenopausal breast cancer cases [125]. Furthermore, one work considered the relationship between the possibility of developing breast cancer and obesity. The data revealed that too low a body weight over 10 to 20 years results in a lower risk of developing breast cancer in women (odds ratio (OR), 0.70; 95% confidence interval (CI), 0.56–0.88, and OR, 0.74; 95% CI, 0.59–0.93, respectively) [126,127]. One study found that women with a high body weight above a BMI of 30 kg/m2 and postmenopausal women had poorer disease-free survival (hazard ratio (HR), 1.43; 95% CI, 1.11–1.86) and overall survival (HR, 1.56; 95% CI, 1.14–2.14) in comparison with women with a normal weight [128].

### 3.2. Diet

Many researchers have placed the association between diet, food, and cancer at the center of their studies. Overweight and obesity have a strong link with the development of breast cancer, and for this reason, it is essential to follow a healthy diet rich in fruit, vegetables, and cereals and low in red meat and saturated fats [129,130]. Data from some studies have shown that a diet free of saturated and polyunsaturated fats can decrease the possibility of developing breast cancer or its recurrence in the case of a post-diagnosis diet [131,132]. Numerous studies have linked excessive use of meat and processed meat to an improved risk of developing BC. The findings show an 11% higher risk of BC for an approximately 10% enhancement in the consumption of processed foods in the diet [133]. A study highlighted that high consumption of red meat is associated with a higher risk of invasive BC, while higher consumption of white meat, such as poultry, compared with red meat, induces a reduction in the risk of developing this disease [134]. In contrast, a diet rich in fruit, cereals, legumes, vegetables, and proteins is linked with a 5% decrease in the possibility of BC developing (HR 0.95) with a follow-up of 19.6 years [135]. In general, a diet that contains elevated quantities of vitamin D, fiber, n-3 PUFA, phytoestrogens, and folic acid is recommended to decrease the possibility of generating breast cancer [136]. It has been shown that the Mediterranean diet, particularly associated with olive oil, is responsible for a reduction in mortality and the incidence of breast cancer [137]. Furthermore, numerous works have demonstrated that bioactive components, such as polyphenols and carotenoids, present in fruits and vegetables may present anti-tumor effects, including regarding breast cancer [138]. The consumption of green tea has been shown to have a positive effect on the prevention of breast cancer. This positive effect is thought to be due to the presence of polyphenols, such as (-)-epigallocatechin-3-gallate (EGCG), with an antioxidant and antiproliferative effect on tumor cells [139,140]. Although studies on the relationship between coffee consumption and BC still conflict, recent studies have shown that caffeine intake induces a reduction in the possibility of pathologies such as BC [141]. Further, increased coffee intake after BC diagnosis has been linked to improved BC and general survival in woman BC survivors [142,143]. 

### 3.3. Vitamin D

Another important factor concerns vitamin D. Vitamin D is found in foods such as fatty fish, red meat, and dairy products but is also produced in the skin during exposure to sunlight by its precursor, 7-dehydrocholesterol [144]. Vitamin D has numerous anti-tumor effects on breast cancer, such as reducing cell proliferation, reducing angiogenesis and invasion capacity, inducing apoptosis, and the differentiation of tumor cells [145].

In fact, numerous studies confirm that the intake of vitamin D in subjects with a deficiency or levels below optimal ones induces potentially protective effects on the growth of BC [146,147]. The results of clinical work have revealed that women with a low serum 25(OH)D level have a 27% greater risk of experiencing this disease compared with women with optimal levels [148]. Instead, it has been shown that high serum levels of 25(OH)D in pre- and postmenopausal women are linked with a decrease in the incidence and mortality rate of BC [149,150]. A study carried out by McDonnell et al. highlighted that women with a serum level of 25(OH)D greater than or equal to 60 ng/mL have an 82% lower incidence rate compared with women with an insufficient serum level. Therefore, high concentrations of 25(OH)D result in a lower risk of breast cancer [151].

These results have been further confirmed by other studies that highlight that serum levels lower than optimal increase the risk of developing breast cancer and increase the risk of death from the disease; conversely, optimal levels reduce these risks. Likewise, vitamin D supplementation plays an important role in preventing the development of neoplasia [152,153]. A further study highlighted that supplementing foods with vitamin D could potentially reduce breast cancer mortality by 10% [154]. Another study demonstrated that supplementation induces a 13% reduction in mortality but demonstrated no effects on incidence [155].

### 3.4. Physical Activity

Although the mechanism is not yet completely clear, physical activity has been shown to be a protective behavior against the onset of breast cancer [156,157]. This important factor is not just restricted to women with a family history of BC but also affects all women both premenopausal and postmenopausal [158]. There are numerous possible mechanisms underlying this action, such as a reduction in exposure to endogenous sex hormones, an action on low-level chronic inflammation, an alteration of the immune system response, and a reduction in oxidative stress [159,160]. Although an optimal level of physical exercise that enables the prevention of BC has not yet been identified, moderate to intense physical activity presents the best results, as confirmed by the WCRF [11,117]. Data obtained in clinical work on postmenopausal women (between 50 and 79 years) have revealed that more intense and continuous physical exercise is correlated with a reduction in the possibility of BC developing (relative risk (RR), 0.86; 95% CI, 0.78–0.95) [161]. Further studies have found that women with BC who perform continuous exercise throughout the week, including moderate-speed walking, have a reduced risk of cancer death [162,163,164]. 

### 3.5. Alcohol Consumption

Numerous works have highlighted the association between alcohol use and the increased danger of BC [123,165]. A possible explanation is given by the increase in estrogen levels and, therefore, hormonal imbalance following alcohol intake but also by the toxic effects of alcohol metabolites [166]. A study has highlighted that alcohol intake increases the risk of estrogen-positive BC [167,168]. Data from the 2015 EPIC (European Prospective Investigation into Cancer and Nutrition) study showed that consuming alcohol at a young age, before the first pregnancy, significantly increases the risk of developing BC [169], inducing morphological alterations to the breast tissue, predisposing it to the development of this disease [158,170]. Furthermore, studies have shown that consuming three to six drinks of wine per week increases the danger of experiencing BC by 15%, while women who drink at least two glasses per day have a 50% greater possibility than non-drinkers. Recent studies suggest that not drinking alcohol almost eliminates the risk of BC [171,172]. 

### 3.6. Smoking

It has been demonstrated that active and passive cigarette smoking can induce pre-carcinogenic events through an increase in possible mutations in tumor suppressor genes and oncogenes [173]. As with alcohol, smoking at a very young age also increases the danger of developing BC; some investigations have revealed that active smoking (hazard ratio (HR), 1.16; 95% CI, 1.00–1.34), passive smoking, and prenatal smoking (HR, 1.18; 95% CI, 1.10–1.27) increase the probability of developing the disease [174,175,176,177]. One study found that passive smoking in a couple contributes to an increased possibility of ER+/PR+ double-positive BC (OR, 1.46; 95% CI: 1.05–2.03; *p* = 0.027) [178]. Ultimately, encouraging the population to adopt a smoke-free lifestyle is essential for the prevention of BC morbidity and mortality.

## 4. Lifestyle and Colorectal Cancer

Colorectal cancer (CRC) is the third most typically diagnosed type of cancer worldwide and the second highest reason for cancer-related death, with approximately 1.9 million new patients and 0.9 million deaths in 2020 (9.4% of cancer-related deaths) [2,35]. Cases of CRC are expected to increase dramatically by 2030, with the most significant increase appearing in underdeveloped nations [179]. Given the increase in the number of screenings, the incidence in adults aged 50 years and older is decreasing; however, it is increasing in those aged under 50 years [180,181]. CRC is a multifactorial condition induced by the wild proliferation of colon epithelial cells. There are several factors responsible for its pathology, including environmental factors, genetic factors such as family history, and chronic inflammation, but also factors that include diet and lifestyle [182,183].

### 4.1. Overweight and Obesity

Overweight (BMI ≥ 25–30 kg/m^2^) and obesity (BMI ≥ 30 kg/m^2^) are modifiable factors correlated with a high danger of developing CRC. Recent reviews have indicated that there is an approximately 18% greater risk for overweight individuals and 32% for obese individuals to develop CRC compared with normal-weight individuals [184,185]. As further confirmation, it is thought that for every 5 kg enhancement in weight, the possibility of developing the disease increases by 3% [186], while one meta-analysis showed that an increase in BMI of 8 kg/m^2^ increases the risk of developing this disease by 10% [187]. Furthermore, even a 2 cm increase in waist circumference is linked with a 4% increased danger of colorectal cancer [188,189]. At the same time, some studies suggest that losing weight may decrease the possibility of CRC developing [190]. Despite numerous data and proof confirming a clear connection between excessive weight and colorectal cancer incidence [191], the molecular pathways that determine this association are poorly understood and are the subject of further studies. From the studies that have been carried out, it emerges that, since adipose tissue is fundamental in the management of energy and inflammation, the generalized inflammatory state of adipose tissue in obesity is one of the main mechanisms that link obesity to the risk of developing CRC [189]. Excessive fat accumulation has been shown to disrupt hormonal homeostasis and cytokine release. Adipose tissue from overweight or obese people produces mediators such as leptin, resistin, TNF-α, IL-1, IL-6, IL-7, and IL-8, important mediators that promote epithelial cell proliferation, block apoptosis of cells, induce oxidative stress, arrest the immune response, induce high levels of insulin-like growth factor-1 (IGF-1), and increase cell proliferation; it also induces insulin resistance or hyperinsulinemia and causes DNA damage [192,193].

### 4.2. Diet

A fundamentally important modifiable factor regarding the danger of CRC developing is diet. Based on numerous studies and data, it has been shown that consuming red and processed meat regularly throughout the week increases the possibility of CRC development by 20–30% [186]. Other works have shown that consuming five portions of red meat per week increases the risk of CRC by 13% [187], while the possibility of developing the disease can grow by approximately 17% for every 100 g of red meat and by around 18% for every 50 g of processed meat consumed per day. One recommendation is to consume no more than 500g per week or 70g per day of red meat [194,195]. However, the molecular mechanisms underlying this carcinogenic action of red meat are not fully understood. We know that, when cooked, red meat releases carcinogenic substances such as heterocyclic amines (HCA), polycyclic aromatic hydrocarbons (PAHs), and N-nitroso compounds. It is well known that HACs and PAHs are carcinogenic compounds with the ability to induce point mutations, such as substitutions, deletions, and insertions, and, consequently, trigger the carcinogenesis pathway. Likewise, nitrosamines and nitrosamides (NOCs) are carcinogens that induce DNA damage [196,197]. Another significant factor is heme, a porphyrin that contributes to the cancerous alteration of colon cells by increasing oxidative stress and inducing lipid peroxidation in intestinal cells. ROS participate in DNA damage and genetic mutations, while reactive lipid peroxides have a cytotoxic effect on epithelial cells, inducing cell surface damage that causes very high cell proliferation. Hyperproliferation leads to epithelial hyperplasia, which can progress to dysplasia and cancer [198]. At the same time, white meat such as fish and poultry is secure and not linked with colorectal cancer development [199]. It has been shown, however, that nutrition rich in dietary fiber, including fruit, vegetables, and cereals, is connected with a decrease in the risk of CRC by up to 50% [200]. A prospective study reported that fiber consumption from bread and breakfast cereals is linked with a 14% reduced danger of colorectal cancer [199]. Numerous works have determined a recommended dose of 20–30 g of fiber per day [193,201]. This type of diet, in addition to the intake of fiber, enables the intake of numerous active components such as vitamins, polyphenols, carotenoids, and folic acid, which have powerful antioxidant, anti-inflammatory, and anticancer properties [138]. For these reasons, high consumption of fruits and vegetables is an essential factor related to a lower chance of developing CRC [202]. It has been shown that the consumption of carotenoids such as lycopene and β-carotene, particularly abundant in carrots, pumpkins, and tomatoes, are important protective factors that prevent the onset and reduce the risk of CRC [203]. Furthermore, the consumption of green tea has also demonstrated a preventive action on colorectal cancer due to the action of important polyphenols such as catechins and epicatechins [204]. Studies have shown that the consumption of a moderate quantity of red wine protects the individual from the development of CRC thanks to the antioxidant and anti-proliferative action of resveratrol, an important polyphenol belonging to the stilbene class [205]. Some studies, confirmed by WCRF/AICR data, have shown that the intake of high quantities of milk has a defensive effect against CR. It is thought that this effect is due to the calcium present in milk, which is capable of protecting the intestinal mucosa by limiting its carcinogenesis [11,206]. 

### 4.3. Vitamin D

A further component that has revealed positive and beneficial effects against the development of CRC is vitamin D through its action of increasing intestinal calcium absorption but also through various other actions, such as its anti-inflammatory properties, immune function improvement, the prevention of angiogenesis, and the alteration of the expression of genes responsible for the proliferation and apoptosis of intestinal epithelial cells [198]. Vitamin D deficiency is linked to a large increase in the risk of inducing this disease. The suggested calcium intake is 700–1250 mg per day, while a maximum of 50 µg/day of vitamin D is recommended [207]. One study evaluated the association between 25(OH)D levels and early-onset colon cancer and found that a high daily vitamin D intake greater than 450 IU per day resulted in a reduced risk of developing the early-onset disease; furthermore, it also highlighted that the intake of vitamin D through food induced a greater reduction than the use of vitamin D supplements [208]. A meta-analysis study highlighted a chemoprotective role in the development of colorectal cancer. It has been shown that high intake of vitamin D and, subsequently, high circulating levels are inversely proportional to the incidence of colorectal adenoma and colorectal cancer and induce greater survival from CRC. The study demonstrated the essential function of vitamin D in limiting the development of colorectal cancer [209]. A further meta-analysis highlighted that the high intake of vitamin D through food induces a significant 25% reduction in the risk of developing CRC compared with individuals who consume a diet low in vitamin D [210].

### 4.4. Physical Activity

Studies have shown that a determining factor leading to CRC is a sedentary lifestyle without exercise. Numerous results demonstrate an increased incidence of CRC in advanced and growing countries, which could also be attributed to physical inactivity. Studies show that physically inactive individuals are approximately 50% more likely to develop the disease than individuals with regular exercise (OR, 1.49; 95% CI, 1.02–2.16) [211,212]. A recent meta-analysis revealed that constant and intense physical exercise could induce a reduction in the risk of colorectal cancer by 19% and 12%, respectively [213]. Furthermore, studies have highlighted that continuous and daily exercise improves the function of the immune system; reduces inflammation; reduces stress; regulates metabolism; helps balance hormone levels; prevents weight gain; and, consequently, can potentially prevent the growth of colon cancer [198,214]. 

### 4.5. Alcohol Consumption

Among the factors responsible for determining the growth of colorectal cancer and increasing its risks is alcohol intake [215]. A time- and quantity-dependent relationship between alcohol consumption and CRC has been found. The risk of developing colorectal cancer is closely linked to the quantity and length of alcohol intake [186]. It is estimated that drinking at least four glasses/day of alcohol enhances the risk of developing the disease by around 52% while consuming one or two glasses per day increases the risk of CRC by approximately 20%; consuming three drinks increases the danger by about 40% [216]. Furthermore, a study that took into consideration the relationship between individuals who drank more than 40 drinks per week and non-drinkers determined that drinkers had a risk of developing CRC that was more than double that of non-drinkers [217]. It is thought that various mechanisms could demonstrate the ability of alcohol to induce carcinogenesis in the colon. Among the most accepted explanations concern the metabolism of alcohol with the consequent production of metabolites, such as acetaldehyde, capable of inducing carcinogenesis in the colon. These metabolites, in fact, are capable of inducing oxidative stress, lipid peroxidation, DNA adducts, mutations, epigenetic alterations, the inactivation of tumor suppressor genes, hormonal imbalances, and immunological dysfunctions, all conditions that lead to carcinogenesis and CRC growth [206,218,219].

### 4.6. Smoking

It has now been demonstrated that cigarette smoking is a verified but modifiable inductive behavior for the growth of CRC; furthermore, the risk increases in relation to the number of cigarettes smoked [220]. Data from some works have shown that smokers have a 2–3 times greater risk of developing CRC than non-smokers in a quantity- and time-dependent manner of exposure [187,221]. Smoking is even correlated with a reduced CRC-specific survival rate, mainly between smokers [222], and around 12% of CRC deaths are due to cigarette smoking [201]. A possible explanation for this increased risk of developing CRC from cigarette smoking can be attributed to carcinogenic substances present in cigarettes, such as nicotine. It has been shown that these substances induce DNA damage, causing mutations in the epithelial cells of the colon and rectum, which result in their transformation into polyposis, which can evolve into invasive adenocarcinoma [223].

## 5. Lifestyle and Prostate Cancer

According to numerous data, prostate cancer (PCa) is one of the most diagnosed malignant tumors in men in the world after lung cancer, with approximately 1.3 million new cases, and it is the fifth most common reason for death from cancer among men, with an evaluated number exceeding 350,000 deaths in 2018 [1]. It is thought that, in the coming years, the incidence rate will grow to exceed 2.3 million new patients by 2040, and the mortality rate will exceed 700,000 cases, with the highest incidence in advanced countries, mainly in the United States, Canada, Australia, and European Union countries [35,224,225].

Prostate cancer, like most types of cancer, is a multifactorial disease whose etiology is not yet fully understood. Numerous factors are related to an increase in the risk of developing prostate cancer, among which, we find family history, genetic characteristics, ethnicity, and age among the main non-modifiable elements [226,227,228]. In addition to these, many modifiable characteristics are also crucial to the growth of PCa, including smoking, alcohol, excessive body weight, physical inactivity, and an inadequate diet [229]. However, the mechanism through which these factors lead to a greater risk of developing PCa is not yet completely clear. The action of non-modifiable factors combined with modifiable ones is thought to induce dysfunction in the genes that regulate epigenetic processes involved in histone modifications in DNA methylation and non-coding miRNAs [230].

For these reasons, given an ever-increasing number of cases, it would be useful to develop a prevention strategy by focusing on modifiable risk factors.

### 5.1. Overweight and Obesity

The correlation between obesity and PCa has generated much debate, but a certain association has not yet been found despite numerous publications and data. While some works have demonstrated a direct association between obesity and PCa incidence, different studies have failed to establish any association [231]. Furthermore, it was recently found that high body weight in adolescence is negatively related to overall PCa hazard [232,233]. These data add to the uncertainty about the role of obesity on PCa, as recent findings suggest that obesity in younger men may paradoxically serve as a protective factor against prostate cancer [234]. However, the results of one study showed that high weight is related to augmented prostate-specific mortality (HR: 1.19, 95% CI: 1.10–1.28) and all-cause mortality (HR: 1.09, 95% CI: 1.00–1.18) [235]. Furthermore, a meta-analysis and review including 23 studies demonstrated a substantial distinction between subjects with high and normal weight (*p* < 0.001), and 54% of obese subjects were at greater risk than normal-weight subjects [236]. Recently, however, a review of data observed no proof of a relationship between BMI and PCa hazard [234]. Likewise, a meta-analysis study discovered that localized PCa has an inverse linear association with body weight, while advanced PCa has a positive linear connection to body weight [237].

Data from other studies indicate that high body weight affects tumor aggressiveness in the early stages of PCa, as supported by other data demonstrating the relationship between BMI before diagnosis and the risk of fatal PCa [238].

Therefore, because the evidence linking a high estimated weight to a PCa diagnosis with poorer results for PCa subjects is conflicting; further studies are needed to evaluate whether weight loss between PCa survivors presents clear advantages regarding PCa recurrence. However, given the relationship between obesity and other malignant tumors, it is necessary to advise men to achieve and preserve an optimal weight.

### 5.2. Diet

The increasing incidence of PCa cases requires the development of prevention strategies, with particular reference to the adoption of a healthy and balanced diet, to decrease the risk of developing PCa. It has been shown that a dietary model based on a high quantity of saturated fats and low intake of fruit, vegetables, and cereals—such as the American model, which is rich in meat and derivatives—is usually related to a high incidence of prostate cancer and a greater tendency for extreme PCa phases [239,240]. On the other hand, in contrast to these data, the Mediterranean dietary model, with minimally processed foods, appears to be safer. In contrast to the Western dietary model, the Mediterranean model is characterized by the reduced consumption of saturated animal fats and red meat and the greater consumption of foods of vegetable origin [241]. The Mediterranean diet is often related to a very low hazard of cancer in general, particularly for CRC, pharyngeal and esophageal cancer, and prostate cancer. This defensive result is due to the ability of these foods to repress accidental mutations and control the mechanisms of cell proliferation, DNA methylation, and apoptosis [242].

Numerous studies have highlighted that the excessive use of products containing high quantities of saturated fatty acids (SFAs) and trans fatty acids (TFAs) represents a greater possibility of generating PCa in the most dangerous stages [243,244]. On the other hand, higher consumption of n-3 eicosapentaenoic acid (EPA) results in a lower risk of fatal prostate cancer [243]. Furthermore, although there are conflicting and unclear data, the excessive use of processed meat and red meat, particularly meat cooked at high temperatures (grilled/barbecued), also presents a favorable impact on the risk of PCa; in contrast, white meat is not connected with the risk of PCa [245]. This association can be explained in several ways; in particular, one of the ways is linked to the high content of SFA within red and processed meat, while another way is related to the many carcinogens presence, such as heterocyclic amines (HAs) and polycyclic aromatic hydrocarbons (PAHs), which are produced when red meat is cooked at high temperatures [246,247].

For these reasons, a Mediterranean and balanced diet is recommended, with greater consumption of foods of plant origin such as vegetables, fruit, bread, potatoes, cereals, beans, seeds, and nuts [241]. Numerous studies have determined that these products have a defensive outcome regarding the development of PCa. One study evaluated the role of tomatoes and their components, such as lycopene, in PCa, resulting in a reduction in the hazard of PCa developing because this compound exercises antioxidant effects and downregulates the pathways implicated in the inflammatory response [248].

Furthermore, bioactive compounds such as monounsaturated fatty acids (oleic acids) and phenolic antioxidants (phenols and flavonoids) in olives present healthy outcomes. Fruit and vegetables even have a high range of flavonoids, which have antioxidant, antimutagenic, and antiproliferative properties [241]. Omega-3 polyunsaturated fatty acids (PUFAs), found in fish and nuts, have a defensive effect on prostate cancer, slowing cancer growth and advancement [249].

### 5.3. Vitamin D

A factor that has generated conflicting results is certainly vitamin D; in fact, its association with PCa is much less evident than its association with BC and CRC. Some works have highlighted that optimal levels of circulating 25(OH)D induce a reduction in the risk of developing PCa, while other studies have shown a linear relationship between increased circulating 25(OH)D and an increased risk of developing this disease [250,251]. For these reasons, it is important to continue to carry out clinical studies on the relationship between vitamin D and prostate cancer.

### 5.4. Physical Activity

Numerous investigations have analyzed the association between carrying out regular and constant physical activity and prostate cancer risk development. Several studies have associated exercise and physical activity—both moderate and recreational but regular up to long-term professional exercise—with a decrease in PCa risk of between 10 and 30% [252,253]. Physical activity appears to have a protective and healthy function against prostate cancer by preventing the growth and advancement of the disease, inducing epigenetic modifications; regulating the inflammatory and immune system response; optimizing muscle tissue; decreasing oxidative stress and levels of hydrogen peroxide involved in carcinogenesis; and improving, on a general level, the quality of life [254,255]. Contrasting results have also been found, probably because of the different physical activities performed; indeed, it has been demonstrated that intense and long-lasting physical exercise induces a higher risk reduction compared with light exercise [252]. Furthermore, it has been shown that patients who perform vigorous exercise for a longer time have a 25% lower risk of dying from prostate cancer [256]. In a prospective cohort study, surviving men diagnosed with stage II-IV PCa had a longer life expectancy if they performed physical activity consistently [257]. Similarly, men who walked or cycled for more than 20 min per day or exercised for more than 1 h per week, who were diagnosed with localized PCa, had lower mortality rates the longer and more intense the activity was [242]. National physical activity guidelines in the USA recommend that adult men perform more than 150 min/week of medium exercise or, at minimum, 75 min/week of intense exercise to have a health benefit [258].

### 5.5. Alcohol Consumption

One of the most significant behaviors that influence the development of numerous tumors, including PCa, is alcohol intake [259]. However, data regarding the impact of alcohol intake on PCa are conflicting. Some investigations have highlighted a non-significant relationship between alcohol consumption and PCa risk; however, other studies have revealed that more elevated alcohol consumption is related to a higher risk of PCa [96,260]. A 2016 meta-analysis demonstrated an 18% increase in PCa morbidity and mortality by comparing drinkers who consumed more considerable quantities, i.e., more than 65 g per day, with those who did not drink (RR, 1.18; 95% CI, 1.10–1.27) [261]. In contrast, numerous investigations have registered opposing connections between alcohol consumption and the chance of developing advanced or fatal prostate cancer [262,263]. Furthermore, one study highlighted that red wine intake is linked to a decrease in PCa risk, mainly in the deadliest forms [108]. Despite these conflicting data, alcohol is still identified as decisive for PCa development, in particular with high consumption and early exposure, as it is responsible for accelerating tumor expansion and greatly reducing the time of advancement toward metastatic prostate cancer [264]. For these reasons, prostate cancer patients are advised to stop drinking alcohol [259].

### 5.6. Smoking

One of the main factors linked to the onset of many tumors is cigarette smoking. Numerous studies have taken into consideration the relationship between smoking and PCa with conflicting results. No relationships have been found between smoking and the incidence of PCa; however, there is evidence demonstrating that smoking is related to an augmentation of the PCa mortality rate by between 15% and 25% [265,266,267]. The data are also inconsistent because of the assessment of smoking, which appears to be difficult since there are notable variables, including being a smoker when the study is being conducted, the age at starting smoking, the length of the smoking period, the number of cigarettes, the time elapsed since quitting smoking, and the pack/year ratio [268,269]. Furthermore, smoking can generate more severe PCa problems after diagnosis, as smokers may have worse responses to treatment, such as radical prostatectomy and radiotherapy [270]. Smoking has also been linked to more developed cancer phases and more aggressive disease factors [242,271,272].

Numerous works have also analyzed the association between smoking and PCa recurrence or progression, and extensive data demonstrate that men who smoke after diagnosis have a higher risk of recurrence or progression than non-smokers [273,274,275]. In particular, work on localized PCa has shown that individuals who had not smoked for more than ten years had a risk of cancer recurrence comparable to non-smokers; smokers who stopped less than ten years previously had an equivalent higher risk of relapse [274]. However, additional analyses are required to comprehend the exact effect of smoking on prostate cancer. What we can say is that men should be helped to quit smoking to improve PCa-specific prognosis and, more importantly, to improve overall health.

## 6. Conclusions

Cancer represents a truly global health challenge with significant medical and financial demands. Preventive interventions at all levels are of fundamental importance in adhering to disease treatment and preventing progression. In this review, we focused on the relationship between modifiable diet and lifestyle factors and the development of breast, colorectal, and prostate cancer. Numerous modifiable factors related to diet and lifestyle appear to influence the progression of various types of cancer. Consistent non-adherence to several suggested lifestyle behaviors—such as not smoking, preserving a healthy body weight, engaging in regular vigorous exercise, and reducing alcohol consumption or eliminating it—appears to augment the tumor progression risk. The adoption of healthy lifestyles is related to significantly lower risks of cancer morbidity and mortality. It is clear that the adoption of just one correct and healthy behavior is not sufficient in preventing the onset of cancer and having a positive impact on the quality and longevity of life; however, as has been highlighted, adhering to multiple recommended healthy lifestyles induces health benefits and prevents the development of these diseases. Based on the results of numerous studies, it is clear that establishing environments that encourage individuals to engage in healthy and regular behaviors is a global public health priority. Further analyses are required to understand the mechanism through which the adoption of correct lifestyles influences the growth and advancement of cancer.

## Figures and Tables

**Table 1:** The 2018 WCRF/AICR Cancer Prevention Recommendations.

Recommendations	Aims	Advice
(1) Be a healthy weight. Body weight must be kept within a healthy limit, preventing weight increases over the years.	➢ Guarantee the body weight during pre-adulthood is close to the lower limit of healthy adult BMI. ➢ Maintain a weight low within the healthy limit throughout life (BMI 18.5 to 24.9). ➢ Avoid weight increase in adulthood.	➢ Consuming a diet rich in cereals, vegetables, fruit, and pulses such as beans. ➢ Restricting “fast foods” and other processed foods high in fat, starches, or sugars. ➢ Reducing sugar-sweetened drinks. ➢ Participate in sports and physical activity.
(2) Perform physical activity. Perform physical activity as part of daily life, move more, and stop less.	➢ Perform moderate physical activity and observe national guidelines. ➢ Reduced passive routines.	➢ Middle-of-the-roader intensity activities: walking, cycling, family chores, gardening, swimming, and dancing. ➢ High-intensity activities: running, fast swimming, fast cycling, aerobics, and team sports.
(3) Consume a diet rich in cereals, vegetables, fruit, and beans. Make these foods part of your daily diet.	➢ Make a diet that supplies at least 30 g/day of fiber. ➢ Consume matrices containing cereals, vegetables, fruit, and legumes such as beans and lentils, and eat at least five meals of fruits and vegetables a day.	➢ Non-starchy vegetables and fruit of different colors. ➢ Non-starchy roots and tubers (e.g., carrots, artichokes, celeriac (celery root), swede (rutabaga), and turnips). ➢ Cereals (e.g., brown rice, wheat, oats, barley, and rye).
(4) Restrict eating of “fast foods” and processed foods high in fat, starches, or sugars. Reducing these foods helps maintain a healthy weight.	➢ Restrict eating of processed foods high in fat, starches, or sugars	➢ Some high-fat foods can be consumed, such as olive oil, avocados, and nuts, which are essential sources of nutrients. Their consumption has not been connected to weight increase.
(5) Reduce the use of red and processed meats.Consume red meat in small doses, such as beef, pork, and lamb. Eat small or eliminate processed meat.	➢ Do not eat red meat more than three times a week. Try to completely eliminate processed meat.	➢ Concerning breast, colorectal, and prostate cancer, do not completely eliminate red meat because it is a source of nutrients, especially protein, iron, zinc, and vitamin B12.
(6) Reduce the use of sugary drinks.Drink mainly water and unsweetened beverages.	➢ Do not ingest sugary beverages.	➢ To maintain proper hydration, it is very useful to consume water or tea without sugar.
(7) Reduce or avoid alcohol use. For better cancer prevention, eliminate alcohol.	➢ Alcohol increases the risk of cancer; it is advisable to eliminate alcohol consumption.	➢ The risk of cancer is linked to the amount of alcohol ingested, and even small quantities increase the risk of developing various cancers. There are no quantities of alcohol below which the risk does not increase.

BMI, body mass index.
